# Distribution of bacteriocin genes in the lineages of *Lactiplantibacillus plantarum*

**DOI:** 10.1038/s41598-021-99683-1

**Published:** 2021-10-08

**Authors:** Sungmi Choi, Min-gyung Baek, Myung-Jun Chung, Sanghyun Lim, Hana Yi

**Affiliations:** 1 Interdisciplinary Program in Precision Public Health, Korea University, Seoul, Republic of Korea; 2 Department of Public Health Sciences, Graduate School, Korea University, Seoul, Republic of Korea; 3 R&D Center, Cell Biotech Co. Ltd., Gimpo, Republic of Korea; 4 School of Biosystems and Biomedical Sciences, Korea University, 145 Anam-ro, Seongbuk-gu, Seoul, 02841 Republic of Korea

**Keywords:** Bacterial evolution, Bacterial genomics

## Abstract

*Lactiplantibacillus plantarum*, previously named “*Lactobacillus plantarum*,” is found in a wide variety of environments exhibiting a high level of intraspecies genetic diversity. To investigate the strain diversity, we performed comparative genomic analyses of the 54 complete genome sequences. The results revealed that *L. plantarum* subsp. *plantarum* was split into three lineages, A, B and C. Of the genes beneficial for probiotic activity, only those associated with the biosynthesis of plantaricin (Pln), an *L. plantarum*-specific bacteriocin, were found to be significantly different among the lineages. The genes related to the biosynthesis of *plnE/F* were conserved throughout the three lineages, whereas the outgroups did not possess any Pln-producing genes. In lineage C, the deepest and ancestral type branch, *plnE/F* genes, were well conserved. In lineage B, loss of gene function was observed due to mobile elements in the *pln* loci. In lineage A, most strains were predicted to produce more than one type of Pln by possessing diverse Pln-encoding genes. These results showed the presence of functional diversity arising from the trifurcating evolution in *L. plantarum* subsp. *plantarum* and demonstrated that Pln is an indicator for differentiating the three lineages.

## Introduction

“*Lactobacillus plantarum*” was reclassified as *Lactiplantibacillus plantarum*^1^ and currently encompasses two subspecies, namely, *L. plantarum* subsp. *plantarum* and *L. plantarum* subsp. *argentoratensis*. *L. plantarum* is widely used as a probiotic because of its health benefits and the history of safe use. Because *Lactiplantibacillus* is present in the normal human flora and aids in the control of pathogens, its application in the development of microbiome therapy products has increased^2^. *L. plantarum* is used in the fermentation of vegetables and dairy products and is found in a variety of fermented products worldwide. As *L. plantarum* is found in diverse habitats, it has evolved in diverse ways, and as a result, a high level of intraspecies genetic diversity is found in this species^3^. Diversity in strains may be advantageous for industrial applications but is disadvantageous with regard to the safety of food products. Comparative gene analysis is currently being used to further investigate this thoroughly.

According to previous studies, the *L. plantarum* genome (3.3 Mb) is larger than the typical genomes of other lactic acid bacteria (LAB; 2–2.7 Mb). The large genome size of *L. plantarum* suggests a very high level of intraspecies genetic diversity, and this characteristic is thought to be because of the nomadic lifestyle of this species, which lives in a variety of habitats and has a large metabolic diversity^4,5^. Owing to the high level of intraspecies diversity, it is not easy to categorize *L. plantarum* strains based on simple traits. Previous comparative genomic analysis studies have consistently demonstrated that the evolution of *L. plantarum* is not related to the isolation source or the geographical location of the strains belonging to this species^5,6^. However, differences in few gene clusters have been found among *L. plantarum* strains. Siezen et al. analyzed six strains in 2011 and reported large differences in prophages, IS elements, transposases, and plantaricin (Pln) biosynthesis genes among the strains and found high variability in capsular polysaccharide and extracellular polysaccharide biosynthesis genes^7^. In 2016, Martino et al. analyzed the presence/absence of orthologous genes using 54 strains and demonstrated that *L. plantarum* strains separated into two phenotypic clusters based on their extracellular polysaccharides, secretome, and sugar metabolism^5^. In 2018, Choi et al. showed that 108 strains could be classified into five phenotypic clusters based on their carbohydrate utilization, virulence, and metabolism^6^. A comparison of 23 strains by Yu et al. in 2017 showed that few strains have evolved to contain a clustered regularly interspaced short palindromic repeat region, antimicrobial activity, and detoxification activity^8^. However, most of these studies classified the strains into phenotypic clusters based on certain functional genes; therefore, they did not examine the gene clusters based on phylogenetic relationships.

Bacteriocins are narrow- or broad-spectrum antimicrobial peptides produced by wide range of prokaryotes and are classified into several categories. Many LAB produce bacteriocins which can be applied to food preservation or food safety applications^9^. Recently, bacteriocins are predicted to have a positive effect on the human intestine by maintaining the balance of bacteria in the gastro-intestinal tract microbiome and affecting the host immune system, which may be of value in disease treatment and health improvement^10^. *Lactiplantibacillus plantarum* is known to produce class I and II bacteriocins, including Plns A-Y, NC81F, NC8HK, and NC8βα^11^. A new *L. plantarum* strain harboring genes coding for different bacteriocins rather than conventional plantaricins and showing broad inhibitory spectrum is discovered recently^12^. The bacteriocins produced by *L. plantarum* are known to reduce the gastrointestinal diseases by inhibiting the growth of pathogens like as *Staphylococcus aureus* and *Listeria*^13^ and also known to possess antagonist effects against food spoilage^14^. Thus, the production of bacteriocin by *L. plantarum* is one of the important criteria for probiotic strain selection.

The present study aimed to investigate the evolutionary trend in *L. plantarum* from a phylogenetic perspective. We first performed phylogenomics analysis to subdivide the species *L. plantarum* subsp. *plantarum* solely on the basis of phylogenetic relationships and aimed to determine whether certain functional genes can be used to differentiate among the intraspecies gene clusters. Using only complete genome sequences, we attempted to minimize the analytic errors that occur from the use of low-quality draft genomes. We focused on a variety of functional genes, including those associated with carbohydrate utilization and substance biosynthesis, to determine whether there is a significant difference between the groups and to predict whether this difference can affect the role of each subgroup as a probiotic.

## Results

### General characteristics of the *L. plantarum* subsp. *plantarum* genomes

Examination of the complete genomes of 54 *L. plantarum* subsp. *plantarum* strains revealed that the genome size ranged from 2.95 to 3.70 Mb, G + C content ranged from 44.08 to 44.93%, and the number of coding sequences (CDSs) ranged from 2729 to 3478 (Table 1). Of the 54 strains studied, 41 contained plasmids in addition to the chromosomes. The core genome consisted of 2207 orthologous gene families, and the pan-genome consisted of 7323 orthologous gene families (Supplementary Fig. S1A). Estimation of openness based on the Heaps’ Law model showed that the *L. plantarum* subsp. *plantarum* pan-genome was open with a parameter (γ) of 0.66 (Supplementary Fig. S1B). It was estimated that approximately 40 new gene families will be added to the pan-genome each time new genomic information on this species is added.

**Table 1:** List of the complete genome sequences analyzed in this study.

Strain	Assembly	Country	Isolation source	Predicted CDSs	Genome size (bp)	GC ratio
LP2	GCA_002109425.1	China	Fermented vegetable	3015	3,284,622	44.55
ST-III	GCA_000148815.2	Korea	Fermented kimchi	3019	3,307,936	44.48
PC520	GCA_002576835.1	China	Fermented vegetable	3209	3,452,904	44.33
CAUH2	GCA_001617525.1	China	Fermented vegetable	2989	3,274,625	44.52
LZ95	GCA_001484005.1	China	Infant feces	3053	3,322,458	44.47
dm	GCA_002220175.1	USA	Drosophila melanogaster	3081	3,325,676	44.52
BDGP2	GCA_002290185.1	USA	Drosophila melanogaster	3362	3,581,586	44.2
KP	GCA_001704315.1	Canada	Drosophila melanogaster	3466	3,692,742	44.11
DF	GCA_001704315.1	Canada	Drosophila melanogaster	3478	3,697,306	44.08
KC28	GCA_002948215.1	Korea	Fermented vegetable	3042	3,291,849	44.5
RI-113	GCA_001990145.1	Switzerland	Fermented salami	3226	3,462,990	44.32
DSM 20174^T^	GCA_014131735.1	Unknown	Fermented cabbage	2968	3,250,154	44.50
TMW 1.1623	GCA_002117245.1	Germany	Fermented food	3061	3,332,882	44.35
JBE245	GCA_001596095.1	Korea	Fermented soybean	2987	3,262,611	44.48
TMW 1.25	GCA_002117245.1	Germany	Fermented sausage	3112	3,351,899	44.3
TMW 1.277	GCA_002117245.1	Germany	Fermented wine	3164	3,400,131	44.22
LM1004	GCA_002895245.1	Korea	Fermented vegetable	2937	3,198,690	44.59
5–2	GCA_001278015.1	China	Fermented soybean	2963	3,237,652	44.66
Zhang-LL	GCA_001581895.1	China	Fermented rice	2729	2,952,218	44.93
LQ80	GCA_003097595.1	Japan	Pig feed plant	3170	3,447,624	44.34
SRCM100434	GCA_002174195.1	Korea	Fermented food	2907	3,223,596	44.6
TMW 1.708	GCA_002117245.1	Germany	Fermented sausage	3001	3,246,485	44.56
10CH	GCA_002005385.2	UK	Fermented cheese	3012	3,311,056	44.51
TS12	GCA_001908455.1	Malaysia	Fermented tofu	3283	3,433,628	44.3
WCFS1	GCA_000203855.3	England	Human saliva	3053	3,348,624	44.42
ZS2058	GCA_001296095.1	China	Fermented vegetable	2900	3,198,337	44.66
B21	GCA_000931425.2	Vietnam	Fermented sausage	3030	3,310,674	44.42
BLS41	GCA_002116955.1	Korea	Fermented vegetable	3214	3,476,011	44.19
CBT LP3	GCA_002286275.1	Korea	Fermented kimchi	3035	3,329,954	44.44
MF1298	GCA_001880185.1	Norway	Fermented sausage	3272	3,564,579	44.22
K259	GCA_002868775.1	Korea	Fermented vegetable	3098	3,373,076	44.49
LB1-2	GCA_002906875.1	Philippines	Apis mellifera	3297	3,541,869	44.14
DOMLa	GCA_000604105.1	China	CDC	2934	3,210,111	44.64
JDM1	GCA_000023085.1	China	Unknown	2923	3,197,759	44.66
ATCC 8014	GCA_002749655.1	Unknown	Unknown	3052	3,309,473	44.47
KC3	GCA_002868755.1	Korea	Fermented vegetable	3123	3,330,006	44.55
GB-LP1	GCA_002220815.1	Korea	Fermented food	2794	3,040,388	44.87
X7021	GCA_002943545.1	China	Fermented tofu	3201	3,407,054	44.38
LPL-1	GCA_002205775.2	China	Fermented fish	2942	3,200,572	44.64
JBE490	GCA_002109405.1	Korea	Fermented rice	2994	3,196,967	44.6
ZJ316	GCA_000338115.2	China	Infant feces	3068	3,299,755	44.49
LZ206	GCA_001659745.1	China	Cow milk	3103	3,263,715	44.55
LZ227	GCA_001660025.1	China	Cow milk	3230	3,425,292	44.35
C410L1	GCA_001874125.1	China	Environment (mud)	3182	3,392,777	44.43
16	GCA_000412205.1	England	Malt production steep water	3076	3,361,015	44.34
P-8	GCA_000392485.2	China	Fermented vegetable	3027	3,246,630	44.55
HFC8	GCA_001302645.1	India	Human feces	3282	3,405,709	44.31
LY-78	GCA_001715615.1	China	Fermented vegetable	2866	3,128,783	44.74
K25	GCA_003020005.1	China	Fermented milk	3176	3,412,154	44.38
CGMCC 1.557	GCA_001272315.2	China	Fermented vegetable	3037	3,273,239	44.44
HAC01	GCA_003143915.1	Korea	Fermented vegetable	2953	3,230,597	44.54
CLP0611	GCA_002024845.1	Korea	Environment	2954	3,230,754	44.54
SRCM102022	GCA_002173655.1	Korea	Fermented food	3084	3,331,364	44.43
NCU116	GCA_001672035.1	China	Fermented vegetable	3090	3,354,689	44.36

The 54 genome sequences of *Lactiplantibacillus plantarum* subsp. *plantarum* available in the public database were included in this study. The type strain genomes of *L. plantarum* subsp. *argentoratensis* DSM 16365^T^ (GCA_003641165.1) and *L. paraplantarum* L-ZS9^T^ (GCA_001443645.1) were used as outgroups but are not presented in this table. The strain information was obtained from the biological descriptions provided in the NCBI database.

### Trifurcating evolution of *L. plantarum* subsp. *plantarum*

From the 56 genomes studied, including the outgroup, 1884 orthologous single-copy core gene sets were extracted. These were concatenated into a sequence comprising 553,499 amino acids, and the maximum-likelihood tree for the sequence was constructed using the Jones–Taylor–Thornton model. Phylogenetic analysis showed a trifurcation-pattern evolution of *L. plantarum* subsp. *plantarum* into lineages A, B, and C (Supplementary Fig. S2). Lineage A consisted of 32 genomes, lineage B consisted of 15 genomes, and lineage C consisted of 7 genomes.

### Independent horizontal gene transfer of the three lineages

**Figure 1:**
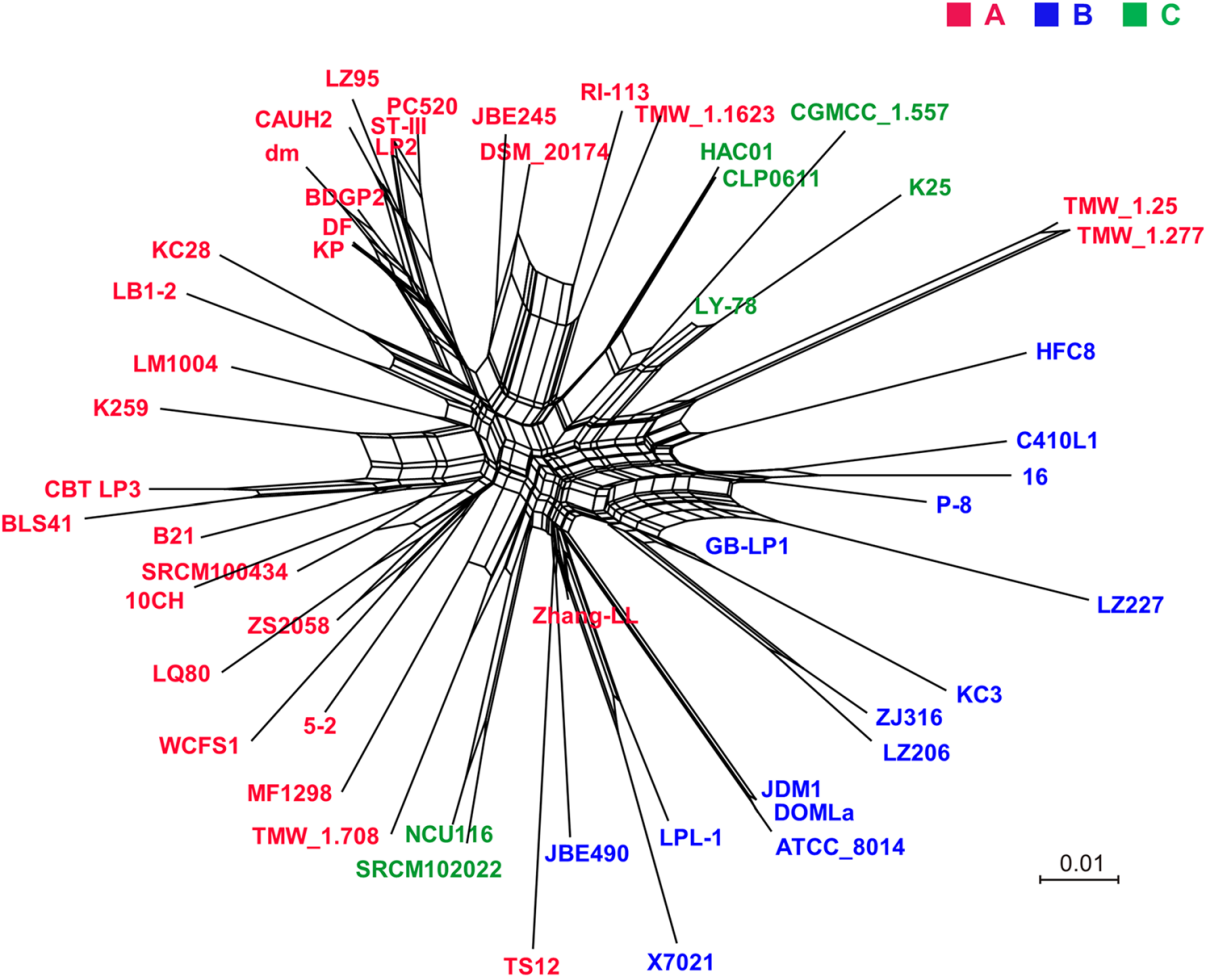
Network tree of *Lactiplantibacillus plantarum* subsp. *plantarum* showing the possibility of nonvertical evolution among strains. The network tree was generated using the SplitTree 4 program based on a binary matrix of the presence and absence of gene families generated using the Ortho-MCL program. Reticulations in the tree indicate the possibility of horizontal gene transfer between branches. The red, blue, and green letters indicate the strains belonging to lineages A, B, and C, respectively.

To evaluate the occurrence of nonvertical evolution in this species, a network tree was generated using a binary matrix in the presence or absence of orthologous genes. The analysis showed that the three lineages were also differentiated in the network tree (Fig. 1). Except for few strains, most strains in each lineage showed intra-lineage levels of horizontal gene transfer, supporting the theory of trifurcating evolution in *L. plantarum* subsp. *plantarum.*

### Differences in bacteriocin biosynthesis among the three lineages

**Figure 2:**
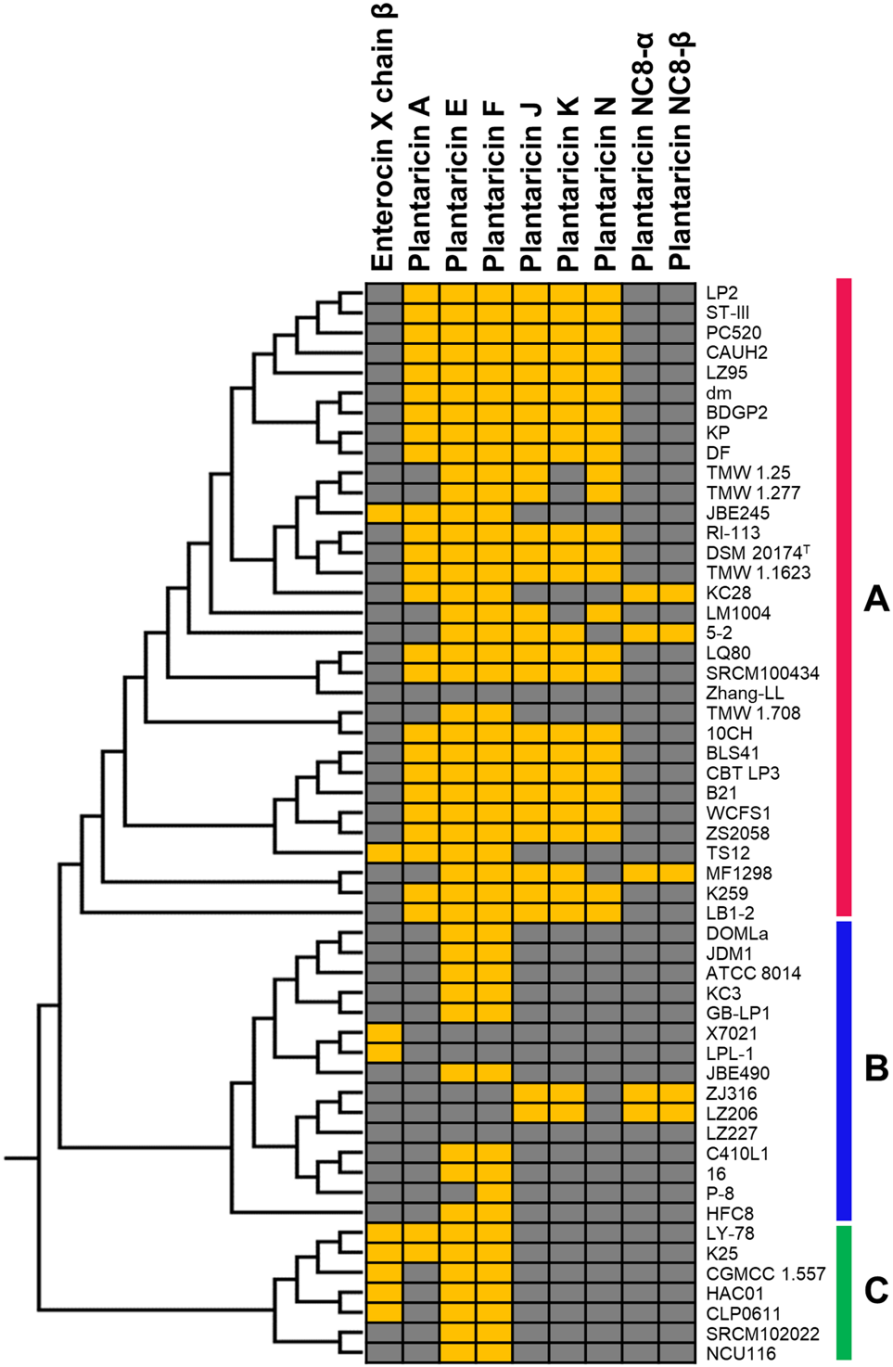
Bacteriocin profiling on the chromosomes of *Lactiplantibacillus plantarum* subsp. *plantarum*. The presence (yellow) and absence (gray) of bacteriocin genes are represented in the matrix. The dendrogram was obtained from the phylogenomic tree constructed using RAxML v8.2.4.

The genes encoding Pln were functional indicators for the trifurcating evolution of *L. plantarum* subsp. *plantarum*. Pln-related genes were only observed in *L. plantarum* subsp. *plantarum*. The sister subspecies, *L. plantarum* subsp. *argentoratensis*, or the outgroup, *L. paraplantarum*, did not possess any Pln-producing genes. Among the diverse *pln* genes identified in *L. plantarum* subsp. *plantarum*, only *plnE/F* genes were observed throughout the three lineages, suggesting that the common ancestor of *L. plantarum* subsp. *plantarum* possessed these genes (Fig. 2).

**Figure 3:**
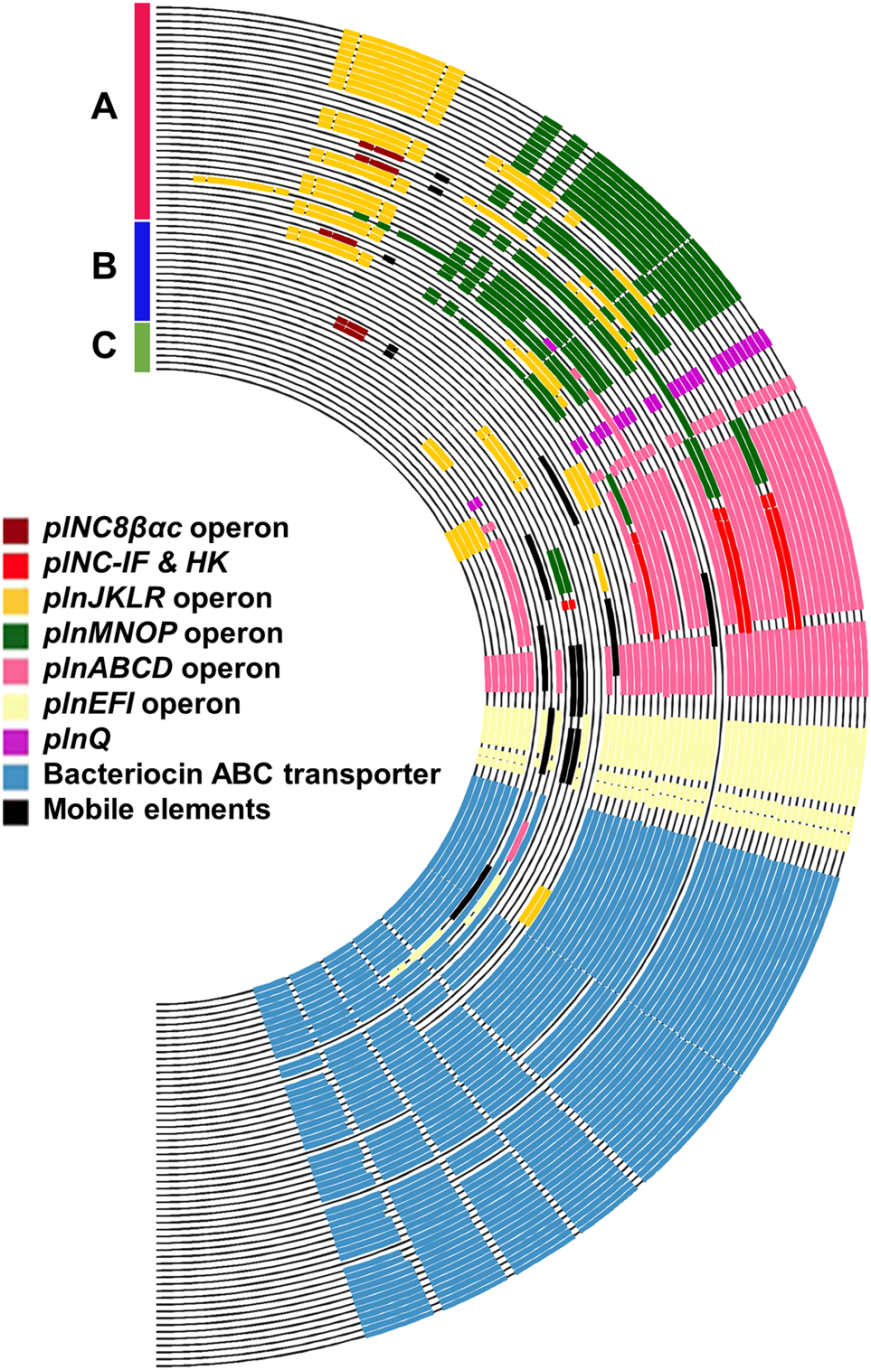
Plantaricin gene (*pln*) loci observed in the 54 complete genomes of *Lactiplantibacillus plantarum* subsp. *plantarum*. The genes related to the biosynthesis and transport systems of plantaricin were analyzed using BLAST and visualized using Circos v0.67. The order of the strains was determined from the results of the phylogenomic tree shown in Supplementary Fig. S1.

In lineage C, the deepest branch, *plnE/F* genes were well conserved in the *plnEFI* operon, and no mobile elements were observed in the *pln* loci of this lineage (Fig. 3). Enterocin X chain β encoding gene was frequently observed in lineage C, but it seemed nonfunctional owing to the lack of chain α-encoding gene. In lineage B, loss of gene function was observed because many *pln* genes were frameshifted, truncated, or disrupted by mobile elements (transposase and integrase). In lineage A, diverse Pln-encoding genes, including *plnA*, *plnQ, plnE/F*, *pln J/K*, and *plnN*, were observed. Comparison of the locations of the Pln operon loci in the genomes showed that the Pln biosynthesis genes were organized into operons and well conserved in lineage A. Except for two strains (Zhang-LL and TMW 1.708), all strains belonging to lineage A were predicted to produce more than one type of Pln.

The plNC8βαc operon^15^, which was found in *L. plantarum* subsp. *plantarum* NC8, was observed in five strains in which the plnMNOP and plnJKLR operons were deleted, and certain mobile element sequences were found. plNC8-IF and histidine kinase (HK) do not encode Pln but control bacteriocin production by encoding induction factor (IF) and HK^16^. The aforementioned five strains, but not the other strains, possess the plNC8βαc operon as well as both IF and HK genes. Unlike the *pln* genes, the plnNC-8 α/β operon and plNC8-IF and HK seemed not originate from a common ancestor but were likely introduced in the genome by mobile elements during the evolutionary process.

### Characteristics of other bacteriocins

In addition to the abovementioned Plns, a small number of bacteriocin genes were sporadically observed, independent of lineage. These included genes related to plnNC-8 α/β found in *L. plantarum* subsp. *plantarum* NC8 and genes related to enterocin. The plnNC-8 α/β operon was found in five strains, and no difference was found in the frequency among the lineages. In all strains containing the plnNC-8 α/β operon, the plnMNOP and plnJKLR operons were deleted, and few sequences of mobile elements were found downstream of the plnNC-8 α/β operon. This suggests that unlike the *pln* genes, the plnNC-8 α/β operon did not originate from a common ancestor but from a different species and was recently acquired by this species via lateral gene transfer. Moreover, certain strains contained genes related to the biosynthesis of bacteriocins other than Pln, which were present on plasmids instead of their chromosomes (Supplementary Table S1).

### Characteristics of probiotic-associated functional genes

Differences in functional genes of interest associated with the following roles were analyzed among the three lineages: (1) utilization of carbohydrates, (2) utilization of human milk oligosaccharides (HMOs), (3) SCFA biosynthesis, (4) acetoin biosynthesis, (5) vitamin biosynthesis, (6) gamma-aminobutyric acid (GABA) biosynthesis, (7) alcohol degradation activity, (8) antioxidant biosynthesis, (9) non-ribosomal peptide synthetases (NRPS) and polyketide synthases (PKS) biosynthesis, and (10) antibiotic resistance. The results of the analyses showed no differences in these functional genes among the strains of the three lineages.

1. Utilization of carbohydrates.

The utilization of carbohydrates was found to be unrelated to the source of strain isolation. It was also found that there was no difference in carbohydrate utilization among the strains of the three lineages. All strains included in the analysis possessed the complete genes required for glycolysis and the pentose phosphate pathway. The strains did not carry the genes required for performing the tricarboxylic acid cycle, which is in agreement with the general characteristics of the genus *Lactobacillus.* In addition, the strains synthesized d/l-lactate from pyruvate, one of the end products of glycolysis, via the fermentation process and also produced acetate or ethanol. Monosaccharides that were used as a carbon source by all strains included glucose, fructose, mannose, and galactose. Disaccharides utilized by all strains included sucrose, maltose, isomaltose, cellobiose, and lactose, and none of the strains could use trehalose. Beta-galactosidase (EC 3.2.1.23), which hydrolyzes lactose, was produced by all strains.

The utilization of rhamnose/rhamnulose varied depending on the strain (Supplementary Table S2). The utilization of sugar alcohol also varied depending on the strain, but this difference was unrelated to the lineage or source of isolation. *L. plantarum* subsp. *plantarum* carries genes encoding two types of phosphotransferase systems (PTS) for the utilization of sorbitol. PTS type 1 was found in most of the strains, whereas PTS type 2 was only present in 10 strains. l-iditol 2-dehydrogenase (EC 1.1.1.14), which converts sorbitol into fructose, was produced by all strains, except for three strains (ATCC 8014, KC3, and P-8) in lineage B, indicating that most strains can metabolize sorbitol. Additionally, the sugar alcohol mannitol was utilized by all strains.

Three types of orthologous genes of alpha-galactosidase (α-Gal, EC 3.2.1.22), which are required for the conversion of galactinol and melibitol to galactose, were found in the strains studied. Genes encoding α-Gal type 1 were carried by most strains included in the analyses, except for JBE49. Genes encoding type 2 were present in all strains in lineages B and C, but the percentage was less than half in lineage A, whereas the genes encoding type 3 were present in only 33% of strains in lineage B, but not in other lineages. Taken together, all strains carried one or more α-Gal-encoding genes.

2. Utilization of HMOs.

To break down HMOs, glycosyl hydrolases, such as GH2 (α-galactosidase), GH16 (endo-β-1,4-galactosidase), GH18 (endo-β-*N*-acetylglycos aminidase), GH20 (β-hexosaminidase/lacto-*N*-biosidase), GH29 (α-1,3/4-fucosidase), GH33 (sialidase), and GH95 (α-1,2-fucosidase), are required. Our analyses showed that all strains in the present study carried genes encoding the GH2 family. However, genes coding for GH16, GH18, GH29, GH33, and GH95 were absent in all strains. Genes belonging to the GH20 family were carried by some strains of lineages A (81%), B (20%), and C (29%).

3. SCFA biosynthesis.

SCFAs are volatile fatty acids that consist of 2–6 carbon atoms and have been recently reported to be closely related to immune function. All strains analyzed contained the formate *C*-acetyltransferase (EC 2.3.1.54) gene, which synthesizes formate from pyruvate, and the 2-acetate kinase (EC 2.7.2.1) gene, which synthesizes propanoate from oxobutanoate. However, genes related to the biosynthesis of butyrate, isobutyrate, valerate, and isovalerate were not found in any strain.

4. (R)-2-acetoin biosynthesis.

Certain bacteria produce acetoin in the form of an energy storage molecule, which is an aroma compound with flavor-enhancing effects. Pyruvate, the end product of glycolysis, is converted to acetoin via (*S*)-2-acetolactate, an intermediate metabolite, by the enzymes acetolactate synthase I/II/III large subunit (EC 2.2.1.6) and acetolactate decarboxylase (EC 4.1.1.5). All strains contained these two genes, suggesting that they used (*R*)-2-acetoin as an energy storage molecule. Additionally, strains BDGP2 and HFC8 synthesized not only (*R*)-2-acetoin but also (*S*)-2-acetoin and contained genes related to meso-butanediol dehydrogenase/(S,S)-butanediol dehydrogenase/diacetyl reductase (EC 1.1.1.-, 1.1.1.76, 1.1.1.304), which converts (*S*)-2-acetoin into (*S*,*S*)-butane-2,3,-diol or meso-butane-2,3,-diol. Strain LZ227 alone utilized an alternative pathway that used the synthesized (*R*)-2-acetoin to produce meso-butane-2,3,-diol and (*R*,*R*)-butane-2,3-diol.

5. Vitamin biosynthesis.

Vitamin biosynthesis in LAB increases their utility as probiotics. The results showed that except for strain WCFS1 in lineage A and strain 16 in lineage B, 51 strains produced thiamin and riboflavin. However, only strain RI-113 in lineage A and strain HFC8 in lineage B synthesized nicotinate. None of the strains synthesized pantothenate, pyridoxal, or biotin.

6. GABA biosynthesis.

GABA is an inhibitory neurotransmitter, and GABAs produced by LAB are used in functional food products. All strains used in this study carried glutamate decarboxylase (EC 4.1.115) genes, which indicates that the strains could produce GABA from glutamate.

7. Alcohol dehydrogenase biosynthesis.

LAB showing alcohol degradation activity are beneficial for alcohol hangover relief and liver health. All strains used in this study carried alcohol dehydrogenase (EC 1.1.1.1) and bifunctional aldehyde-alcohol dehydrogenase (EC 1.2.1.10) genes and therefore had alcohol degradation activity. However, 29–188 amino acids were deleted in the N-terminal region of the protein produced by strain B21 in lineage A and strain LZ206 in lineage B, indicating that these strains could not synthesize the enzyme or that the synthesized enzyme would be inactive.

8. Antioxidant biosynthesis.

LAB strains that remove reactive oxygen species are of interest for industrial applications. All strains used in this study carried genes encoding known antioxidant enzymes, including glutathione peroxidase, glutathione reductase, catalase, and peroxidase (POD). All strains carried only one copy of glutathione peroxidase. Four types of POD (POD types 1–4) genes were present in all strains, whereas type 5 was only found in four strains (HFC8, HAC01, K25, and MF1298).

Glutathione reductase type 1–4-encoding genes were present in all strains. However, strain CBT LP3 in lineage A carried type 5 genes instead of type 4 genes. This was because of a frameshift in the type 4 gene of CBT LP3, which led to the prediction of two CDSs. Glutathione reductase type 6 was found in the plasmids of seven strains (CBT LP3, CAUH2, CGMCC 1.557, LZ206, LZ227, NCU116, and SRCM102022), and no difference was found among the lineages.

9. NRPS-PKS biosynthesis.

NRPS-PKS is a large multienzymatic megasynthase that aids in the defense against pathogenic bacteria by producing a number of antimicrobial substances. Orthologs of the genes related to NRPS synthesis were found in the genomes of strains TMW 1.708, B21, and WCFS1 in lineage A. As gene clusters involved in fatty acid synthesis are present near the genes related to NRPS synthesis, these strains were predicted to be able to synthesize NRPS, and the monomer that can be synthesized was predicted to be (Ala-Ala-Ala-Ser-Gly) + (Ala). The Norine database was used to search for this monomer structure, which was identified as pyoverdine GM, a type of siderophore. Strain LQ80 alone carried two genes (LpLQ80_16120 and LpLQ80_16125) containing a fatty acid-NRPS module in a plasmid.

10. Antibiotic resistance.

CARD was used for the analysis, and the results showed that none of the strains used in this study carried genes related to antibiotic resistance.

## Discussion

The present study aimed to investigate the intraspecies functional diversity of *L. plantarum* subsp. *plantarum* by performing comparative genomic analysis of strains isolated from a variety of sources. The results showed that the sizes of the core- and pan-genomes showed a similar pattern to that in previous studies that used draft genomes for the analysis^5,6^. Genomic and phylogenetic analyses supported the trifurcation of *L. plantarum* subsp. *plantarum* strains into lineages A, B, and C. The three lineages could not be clearly differentiated by the geographical location of the strains (Supplementary Fig. S3). However, most strains isolated from Western countries belong to lineage A. For the rest, the geographic localization of the strains was observed in several sub-clades with solid clustering. In certain LAB^17^, the source of isolation correlates with sugar metabolism traits; however, it has no correlation with the species *L. plantarum*^5,6^, and the present study also found similar results.

The only difference among the three lineages was the presence of bacteriocin-encoding genes. The two main plantaricins observed in this study are Pln EF (in lineages A and C) and JK (in lineage A). Both plantaricins form pores in the plasma membrane of target bacteria, but show complementary ion selectivity^18^. Pln EF pores show high conductivity for cations but not for anions, while Pln JK pores conduct anions well but not cations^18^. Thus, the killing efficiency is enhanced in the strains which possess both complementary bacteriocins. This implies the superior adaptation of lineage A to lineage B or C in the competitive microbial community. Loss of gene function due to mobile genetic elements was observed in lineage B, suggesting the gradual loss of genes related to the biosynthesis of bacteriocins. It is hypothesized that the common ancestor of *L. plantarum* subsp. *plantarum* possesses Pln E/F, and this feature is preserved in the deep branch (lineage C). The descendant types further split into two lineages (A and B), and the evolution of these two lineages was clearly differentiated. Lineage A acquired diverse *pln* genes and GH20 genes for adaptive evolution in competitive environments with HMOs, whereas lineage B was adapted to environments where Pln was not required.

Interestingly, the majority (75%) of lineage A was fermented food origin while the majority (60%) of lineage B was environment or intestinal origin. In addition, strains of lineage C were all originated from fermented food sources. It is known that bacteriocin’s critical role in mediating population- and community-level interactions is ensured when the cell density is high in the environment^19^. Bacteria use bacteriocin to compete with close cells in dense population, but they do not need to produce bacteriocin in low cell density environments. Thus, it is presumed that lineages A and C strains have evolved and adapted to high cell density of fermenting foods by possessing bacteriocin genes, while lineage B strains which have adapt to natural environments are losing the unnecessary genes.

This study demonstrated that there was no difference in sugar utilization among the three lineages. A previous study found differences in fructooligosaccharide and raffinose utilization between the two groups of *L. plantarum* that were classified based on a single nucleotide polymorphism phylogenetic tree^6^. Similarly, a study that performed hierarchical clustering based on sugar gene loci reported a difference in the genes related to sugar metabolism among the clusters^5^. However, these studies divided the clusters based on the presence or absence of orthologous genes or sugar utilization genes, whereas the phylogenetic tree in the present study was drawn based on whole genomes. Therefore, it was not possible to directly compare our results with those of these studies.

Minor differences in the utilization of HMOs were found among the lineages. HMOs contain a common lactose at their reducing end, which is elongated with various combinations of *N*-acetyllactosamine (Galβ1-4GlcNAc) or lacto-*N*-biose I units (Galβ1-3GlcNAc) and then fucosylated and/or sialylated to produce approximately 200 types of oligosaccharides. Therefore, a variety of glycoside hydrolase (GHs) is necessary for the utilization of HMOs. More specifically, GH2 (α-galactosidase), GH16 (endo-β-1,4-galactosidase), GH18 (endo-β-*N*-acetylglycosaminidase), GH20 (β-hexosaminidase/lacto-*N*-biosidase), GH29 (α-1,3/4-fucosidase), GH33 (sialidase), and GH95 (α-1,2-fucosidase) are known to be involved in HMO utilization^20^. The results of this study revealed that almost all *L. plantarum* subsp. *plantarum* strains, regardless of their lineage, carried GH2, suggesting their ability to use lactose. In contrast, the GH20 genes were found more frequently in lineage A than in lineage B or C, implying the advanced adaptation of lineage A to HMOs than that of other lineages.

The bacteriocin distribution within a phylogenetic clade has been investigated in several fermenting environments. Gontijo et al. found that the distribution of bacteriocin structural genes is related to phylogenetic clades of LAB species of artisanal cheese, with a higher frequency in some specific clades^21^. Azevedo et al. reported the occurrence of different classes of bacteriocin among phylogenetic clades of ruminal bacteria and archaea^22^. Collins et al. reported the bacteriocin gene diversity and complexity across the *Lactobacillus* genus complex^23^. Those previous studies accounted the interspecific genetic diversity of bacteriocin production genes, but our study focused on the intraspecific distribution within the commercially important species.

The production of bacteriocins by *L. plantarum* is an indicator of their potential use as natural food preservatives and as dietary supplements. Therefore, in terms of Pln production, the strains in lineage A seemed to have better bacterial community control power than that of the strains in lineage B and C. Previous comparative genomic studies on *L. plantarum* have not featured the association between the evolutionary lineages and the source of isolation. The differential distribution of bacteriocin genes and biased ecological origin inherent to the trifurcating lineages imply the habitat adapted evolution occurring in this species.

## Methods

### Genome sequences

Among the *L. plantarum* subsp. *plantarum* genomes available in the GenBank database of the National Centre for Biotechnology Information (Bethesda, MD, USA) as of May 2018, the complete genomes of 54 different strains were selected for comparative genomic analysis (Table 1). *Lactobacillus plantarum* subsp. *argentoratensis* strain DKO 22^T^ was obtained from the German Collection of Microorganisms and Cell Cultures GmbH (DSM 16365^T^), and the complete genome sequence was determined in this study (GCA_003641165.1). The genome of *L. paraplantarum* L-ZS9 (GCA_001443645.1) was obtained from GenBank and used as an outgroup in the phylogenomic analyses.

### Gene prediction and pan- and core-genome analysis

Protein coding sequences (CDSs) were predicted using Prodigal v.2.6.3^24^, and the Rfam database^25^ was used to search for noncoding RNA genes. Orthologous gene families were analyzed using OrthoMCL^26^, which utilizes an all-against-all protein–protein basic local alignment search tool (BLAST) and a Markov cluster algorithm, with an inflation value of 2.0. Pan/core-genome curves were drawn using PanGP v.1.0.1.^27^. The openness of the pan-genome was estimated based on the Heaps’ Law model using the R package micropan^28^.

### Genome-based construction of the phylogenetic tree

A phylogenetic tree was constructed by selecting only single-copy orthologous genes that were included in the core genome. The amino acid sequences of these genes were aligned using MUSCLE v3.8.31^29^. The aligned positions showing a gap in more than 50% of the 54 genomes were removed using Gblocks v0.91^30^. The final sequence alignments were concatenated using FASconCAT^31^. A model test was performed using ProtTest v3.2^32^ to select a suitable evolution model, and a maximum-likelihood tree was constructed using RAxML v8.2.4^33^. A binary matrix of the presence or absence of orthologs was calculated to analyze the degree of non-vertical gene transfer within the groups. SplitTree v4.14.5^34^ was used to construct a network tree. All phylogenetic trees were drawn using Dendroscope v3.2.2^35^.

### Functional annotation of gene families

Functional classifications of the gene families including orthologs and singleton sequence orphan open reading frames were performed through BLAST searches using UniProt^36^, Clusters of Orthologous Groups of proteins^37^, and Pfam^38^ databases. Glycoside hydrolase (GH) family annotation was performed using the Carbohydrate-Active enZYmes database^39^. Metabolic pathways for short-chain fatty acid (SCFA) and vitamin biosynthesis were analyzed using the Kyoto Encyclopedia of Genes and Genomes automatic annotation server^40^. Gene clusters involved in the biosynthesis of non-ribosomal peptides and polyketides (NRPS/PKS) were predicted using antiSMASH 3.0^41^. Nonribosomal peptides were identified using the Norine database^42^. The resistance gene identifier of the Comprehensive Antibiotic Resistance Database (CARD)^43^ was used to identify antibiotic resistance genes.

### Bacteriocin analyses

Bacteriocin-encoding genes were identified using Bagel3^44^. The presence or absence and the location of previously identified bacteriocin genes, including the *L. plantarum*-specific bacteriocin (*pln*) and *Enterococcus*-specific bacteriocin (enterocin), were analyzed. The graphical *pln* loci map of the complete genome was constructed and visualized using Circos v0.67^45^.

## Supplementary Information

Supplementary Information.
