# Supplementary material for: Distribution of bacteriocin genes in the lineages of Lactiplantibacillus plantarum
Source: Sci Rep. 2021 Oct 8;11:20063. doi: 10.1038/s41598-021-99683-1 (PMC8501086; doi:10.1038/s41598-021-99683-1)
Supplement: Supplementary file 1 — Supplementary Information. [file 41598_2021_99683_MOESM1_ESM.docx]

**Figure S1. Pan- and core-genomes of 54 *Lactiplantibacillus plantarum* subsp.** ***plantarum* strains**

(A) Graphs presenting pan-genome (blue) and core-genome (red). (B) New gene family distribution after sequential addition of each genome to the analysis. Figures were generated using PanGP v.1.0.1.


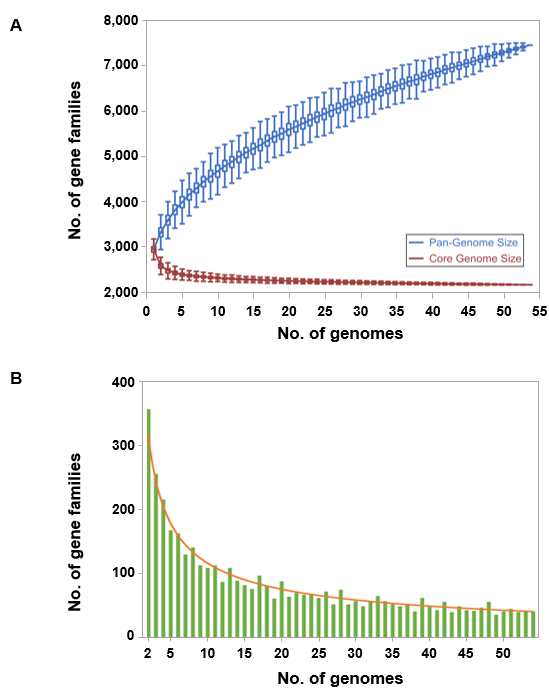


**Figure S2. Maximum-likelihood tree of *Lactiplantibacillus* *plantarum* subsp.** ***plantarum* genomes inferred using amino acid sequences of 1,884 single-copy genes**

Numbers above the branches show bootstrap support from 100 nonparametric replicates. The tree was rooted in *L. paraplantarum* L-ZS9. The tree was constructed using RAxML v8.2.4 and the scale bar represents the number of substitutions per site.

**
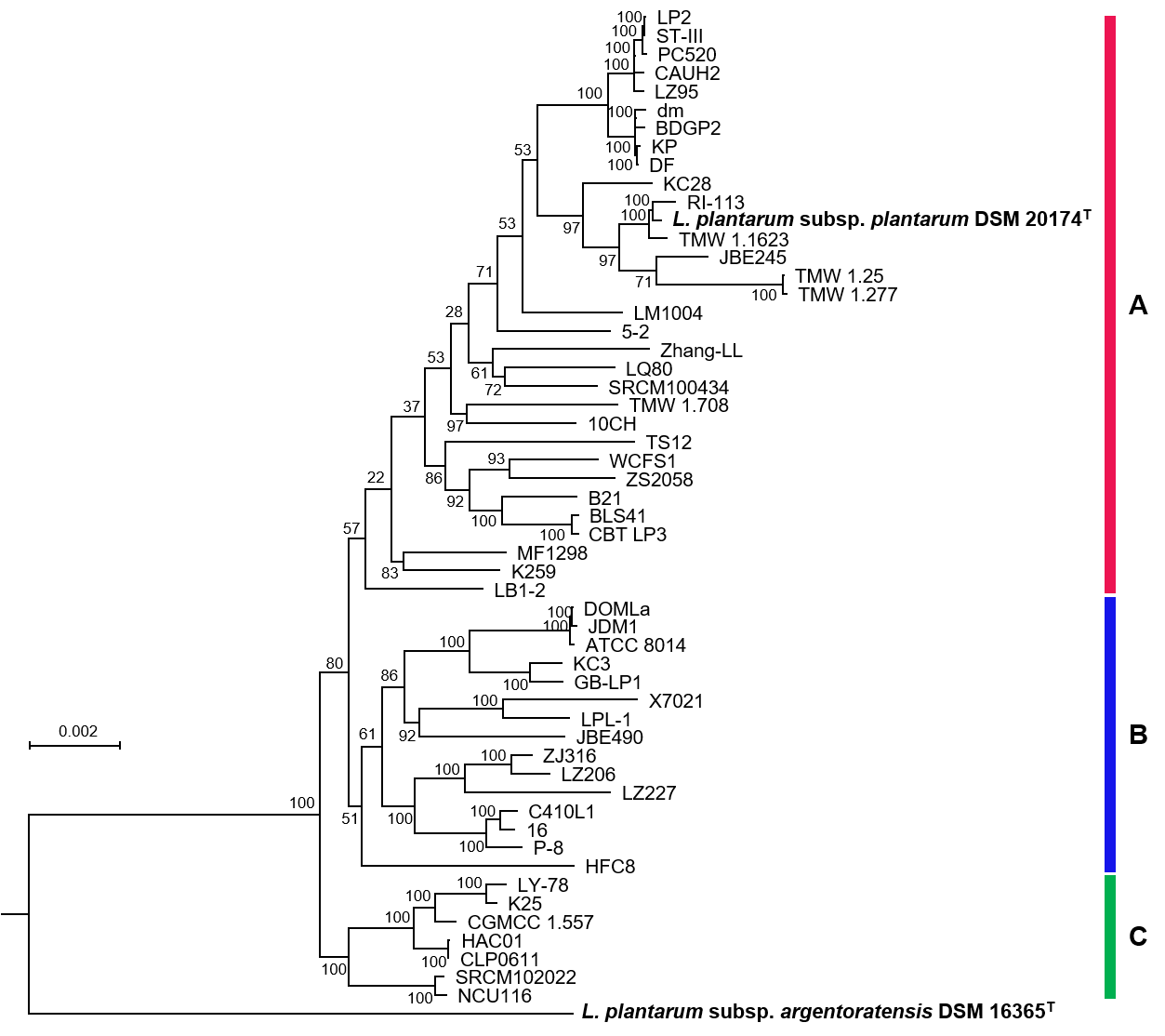
**

**Figure S3. Phylogenomic tree of *Lactiplantibacillus plantarum* subsp.** ***plantarum* indicated with the isolation source and geographical location.** The maximum-likelihood tree was inferred using amino acid sequences of 1,884 single-copy genes.


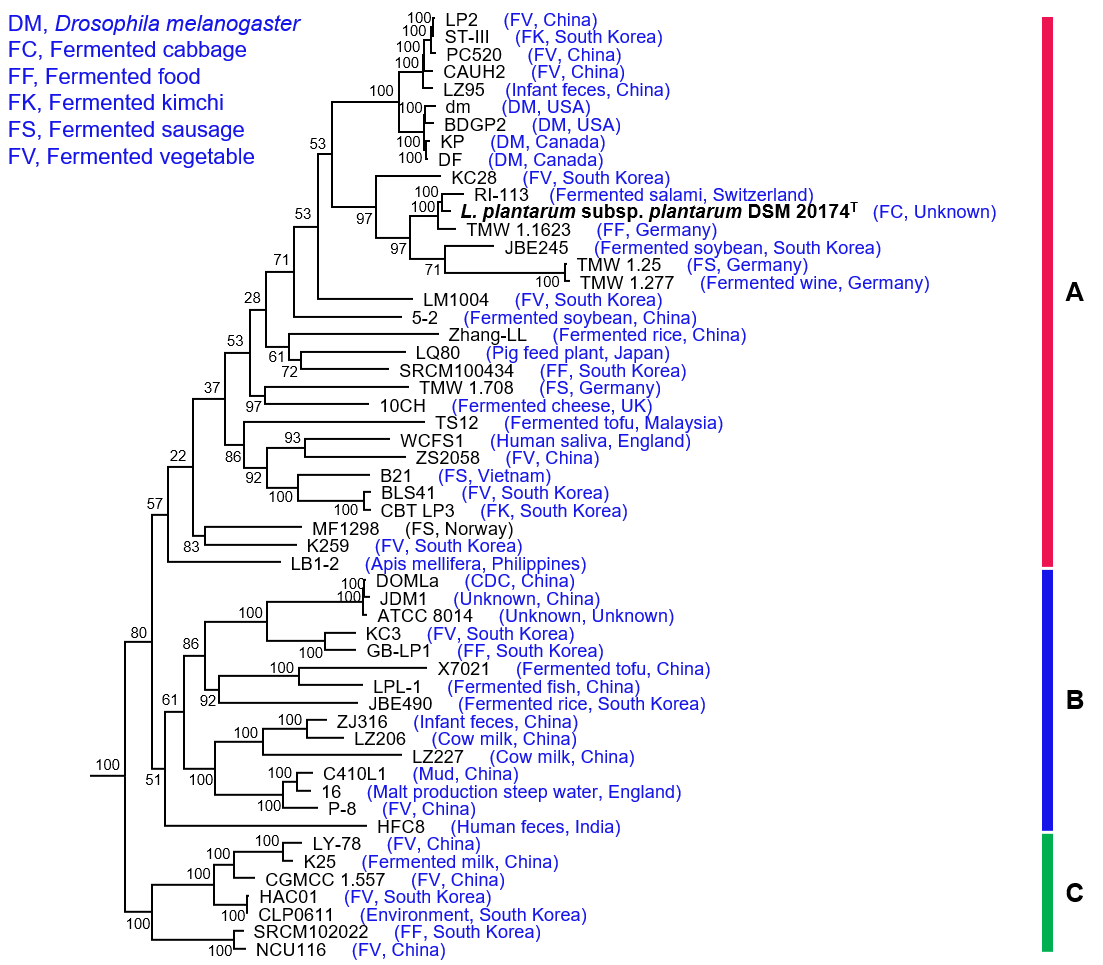


**Table S1. Bacteriocin genes observed on the plasmids of *Lactiplantibacillus* *plantarum* subsp.** ***plantarum***

| **Bacteriocin** | **Lineage A** | | | |  | **Lineage B** | |
| --- | --- | --- | --- | --- | --- | --- | --- |
|  | **PC520** | **TMW 1.25** | **TMW 1.277** | **B21** |  | **LPL-1** | **LZ227** |
| Gassericin A | - | - | - | + |  | - | - |
| Lacticin Z | - | + | + | - |  | - | - |
| Pediocin | + | - | - | - |  | + | + |
| PentocinKCA1 | - | - | - | + |  | - | - |
| Plantaricin 125  beta(plnB) | - | + | + | - |  | - | - |
| Plantaricin 423 | - | - | - | - |  | + | - |
| Plantaricin C19 | - | - | - | - |  | + | - |
| Salivaricin P Cian a | - | + | + | - |  | - | - |

**Table S2. Differences of carbohydrates-utilization gene profile among *Lactiplantibacillus* *plantarum* subsp.** ***plantarum* strains**

| **Lineage** | **Strain** | **Rhamnose/ Rhamnulose** | **Genes for sorbitol utilization** | | | **Alpha-galactosidase orthologue types** | | |
| --- | --- | --- | --- | --- | --- | --- | --- | --- |
|  |  |  | **Sorbitol/**  **glucitol PTS type1** | **Sorbitol/**  **glucitol PTS type2** | **L-iditol 2-dehydrogenase** | **Type1** | **Type2** | **Type3** |
| **A** | LP2 | + | + | - | + | + | - | - |
|  | ST-III | + | + | - | + | + | - | - |
|  | PC520 | + | + | - | + | + | - | - |
|  | CAUH2 | + | + | - | + | + | - | - |
|  | LZ95 | + | + | - | + | + | - | - |
|  | dm | + | + | - | + | + | - | - |
|  | BDGP2 | + | + | - | + | + | - | - |
|  | KP | + | + | - | + | + | - | - |
|  | DF | + | + | - | + | + | - | - |
|  | KC28 | + | + | - | + | + | + | - |
|  | RI-113 | + | + | - | + | + | - | - |
|  | DSM 20174^T^ | + | + | - | + | + | - | - |
|  | TMW 1.1623 | - | + | - | + | + | - | - |
|  | JBE245 | + | + | - | + | + | - | - |
|  | TMW 1.25 | - | + | - | + | + | + | - |
|  | TMW 1.277 | - | + | - | + | + | + | - |
|  | LM1004 | + | - | - | + | + | + | - |
|  | 5-2 | + | + | - | + | + | + | - |
|  | Zhang-LL | - | - | - | + | + | + | - |
|  | LQ80 | + | + | + | + | + | + | - |
|  | SRCM100434 | + | + | - | + | + | + | - |
|  | TMW 1.708 | - | + | - | + | + | + | - |
|  | 10CH | + | + | + | + | + | + | - |
|  | TS12 | - | - | - | + | + | + | - |
|  | WCFS1 | + | + | + | + | + | - | - |
|  | ZS2058 | + | + | + | + | + | - | - |
|  | B21 | + | + | + | + | + | + | - |
|  | BLS41 | + | + | + | + | + | - | - |
|  | CBT LP3 | + | + | + | + | + | - | - |
|  | MF1298 | + | + | + | + | + | + | - |
|  | K259 | + | + | - | + | + | + | - |
|  | LB1-2 | + | + | - | + | + | - | - |
| **B** | DOMLa | + | + | - | + | + | + | - |
|  | JDM1 | + | + | - | + | + | + | - |
|  | ATCC 8014 | + | + | - | + | + | + | - |
|  | KC3 | + | + | - | - | + | + | + |
|  | GB-LP1 | - | + | - | - | + | + | + |
|  | X7021 | + | + | - | + | + | + | - |
|  | LPL-1 | + | + | - | + | + | + | - |
|  | JBE490 | + | - | - | + | - | + | - |
|  | ZJ316 | - | + | - | + | + | + | + |
|  | LZ206 | - | + | - | + | + | + | + |
|  | LZ227 | - | + | - | + | + | + | + |
|  | C410L1 | + | + | - | + | + | + | - |
|  | 16 | + | + | - | + | + | + | - |
|  | P-8 | + | + | - | + | + | + | - |
|  | HFC8 | + | + | - | - | + | + | - |
| **C** | LY-78 | - | + | - | + | + | + | - |
|  | K25 | - | + | - | + | + | + | - |
|  | CGMCC 1.557 | - | + | - | + | + | + | - |
|  | HAC01 | - | + | - | + | + | + | - |
|  | CLP0611 | - | + | - | + | + | + | - |
|  | SRCM102022 | + | + | + | + | + | + | - |
|  | NCU116 | + | + | + | + | + | + | - |
